# The Success of Homœopatic Practice

**Published:** 1853-08

**Authors:** De Laskie Miller


*The Succe**s**s of Homœopathic Practice.*—By DE LASKIE MILLER, M. D.

THERE are, no doubt, *some* persons who believe that the *apparent* success of Homœopathy is the legitimate result of efficiency in their system of practice. While, however, a much larger number of the people entertain the impression, that their boasted success is entirely negative; in other words, without themselves understanding the *difference*, they believe that patients treated according to homœopathy *get well*, instead of *being cured*.

We are satisfied that, if the members of the medical profession would direct more attention to this point in practice, many errors would be avoided, and much of the odium of our profession removed; for this would lead to closer discrimination, and a more judicious course of treatment. More reliance would be placed on the dynamic forces of the system, which, in the phraseology of the older schools, is the "*vis medicatrix Naturæ;*" and the physician would become, as he should be, the conservator of nature. There can be no doubt that the almost unparalleled success which attended the herculean operations of the late Prof. McLelland, was the result of his accurate discrimination, and ability to calculate upon the recuperative powers of nature. A case coming under our own observation is in point.

Some six years since, a young lady, aged 18 years, applied to a physician of respectable standing on account of severe neuralgic pains, affecting different parts of the body in succession. The case was decided to be one of spinal disease, and treated accordingly. For three years this patient was under the most active treatment, such as blisters, setons, and issues; and internally, alteratives, tonics, and anodynes, till she became so far reduced as to walk with great difficulty, and with no amelioration of her symptoms. She then, under the advice of another physician, discontinued all medicine, took regular exercise, and immediately began to improve in strength and spirits, and regained perfect health. Who doubts that in this case the attending physician failed to detect the symptoms caused by the treatment?

Another case. A lawyer of eminence in this city, a few days since, sent a message to an intelligent and experienced physician to visit his wife, who was considered quite sick. The physician went, examined the case, and told the lady that she needed "*rest*" but if she did not feel well by the next day, she might take a blue pill. On the following day the husband called for the prescription of blue pill. "Is your wife sick to-day?" "No; she feels quite well." "Then," replied the doctor, "she has no occasion to take the medicine."

Is it not absolutely certain that, had the first case passed into the hands of a homœopath, it would have been heralded as a triumphant vindication of the efficacy of their system; and that the last would have confirmed the patients in the assumptions of this phase of quackery?

What we would teach is: Always have *clear indications* as the basis for active treatment; or on the maxim of the celebrated Rush—"**When you know not what to do, do nothing.**"

